# Mr. Clarke, on Mixtures with Spermaceti

**Published:** 1800-03

**Authors:** Charles Mansfield Clarke

**Affiliations:** Carey-street


### *To the Editors of the Medical and Phyſical Journal.*

GENTLEMEN,

A Very frequent and common objection made by patients to take medicines containing ſpermaceti, is, that in the uſual way of preparing them, the mixture is not ſmooth and uniform, but lumps of the ſpermaceti ſwim on the ſurface. To remedy this inconvenience, I was induced to try whether it would not mix better if the ſpermaceti was firſt melted, and poured into a mortar, (which ſhould be previouſly warmed,) then a ſufficient quantity of the yolk of eggs to be added, and afterwards the water in the uſual way. This I found to ſucceed perfectly in forming a ſmooth and uniform mixture, without any of the inconveniences above alluded to, and taking up much leſs time than the uſual method of preparing it. By making the above known through the medium of your publication, you will much oblige,

Your humble ſervant,

CHARLES MANSFIELD CLARKE.

